# Clinical and Radiographic Outcomes of Single Implant-Supported Zirconia Crowns Following a Digital and Conventional Workflow: Four-Year Follow-Up of a Randomized Controlled Clinical Trial

**DOI:** 10.3390/jcm13020432

**Published:** 2024-01-12

**Authors:** Florian Beck, Lana Zupancic Cepic, Stefan Lettner, Andreas Moritz, Christian Ulm, Werner Zechner, Andreas Schedle

**Affiliations:** 1 Division of Oral Surgery, University Clinic of Dentistry, Medical University of Vienna, 1090 Vienna, Austria; 2 Division of Prosthodontics, University Clinic of Dentistry, Medical University of Vienna, 1090 Vienna, Austria; 3 Austrian Cluster for Tissue Regeneration, Ludwig Boltzmann Institute for Experimental and Clinical Traumatology, 1200 Vienna, Austria; 4 Core Facility Hard Tissue Research and Biomaterial Research, Karl Donath Laboratory, University Clinic of Dentistry, Medical University of Vienna, 1090 Vienna, Austria; 5 Division of Conservative Dentistry and Periodontology, University Clinic of Dentistry, Medical University of Vienna, 1090 Vienna, Austria; 6 Division for Dental Student Training and Patient Care, University Clinic of Dentistry, Medical University of Vienna, 1090 Vienna, Austria; 7 Competence Center Dental Materials, University Clinic of Dentistry, Medical University of Vienna, 1090 Vienna, Austria

**Keywords:** CAD/CAM, digital dentistry, dental implant, digital workflow

## Abstract

Purpose: This study aimed to compare the clinical and radiographic outcomes of single posterior screw-retained monolithic implant crowns following a digital and conventional workflow and to report on the survival/complication rate after a mean 4-year follow-up. Materials and Methods: Thirty patients with a single posterior tooth missing were rehabilitated with a bone-level implant. After a healing period of ≥3 months, they were subjected to both a digital and conventional workflow to fabricate two screw-retained monolithic implant crowns. The quantitative clinical adjustments to both crowns (intrasubject comparison) and a questionnaire were recorded at try-in. Thereafter, a crown of the digital and conventional workflows was randomly inserted. At the last follow-up, the marginal bone level (MBL), peri-implant health-related parameters (bleeding on probing (BoP), plaque, pocket probing depth (PPD)), and functional implant prosthodontic score (FIPS) were assessed. Furthermore, the implant survival and success rates and technical complications were evaluated. Results: A total of 27 patients were followed for a mean period of 4.23 ± 1.10 years. There was no significant difference between the digital and conventional workflows regarding clinical adjustments and questionnaire outcomes. More than twice as many participants recommended digital (n = 16) compared to conventional impressions (n = 7) to friends. The implant survival and success rate were 100% and 96.3%, respectively. Furthermore, two de-cementations and one fracture of the ti-base abutment occurred. There were no significant differences in BoP, plaque, and PPD metrics between the two groups. The changes in the MBL between implant crown insertion (baseline) and the last follow-up were 0.07 ± 0.19 mm and 0.34 ± 0.62 mm in the digital and conventional groups, respectively (*p* = 0.195). The mean overall FIPS score was 8.11 ± 1.37 (range: 5–10). Conclusions: The clinical and radiographic outcomes of single screw-retained monolithic implant crowns were similar between both workflows after a mean of 4 years of service. The patients did not clearly prefer an impression technique for their restoration, although they would recommend the digital impression more often to friends. Thus, decision regarding clinical workflows may be based on the patient’s and/or clinician’s preference.

## 1. Introduction

Digital workflows have enriched the process of taking impressions of dental implants, as time-demanding steps in the laboratory, e.g., pouring and mounting dental casts, can be omitted, and the virtual design of fixed restorations can be commenced immediately [1,2]. Despite increasing evidence from studies reporting on the accuracy of impressions performed using intraoral scanning (IOS) devices, heterogeneity exists regarding their unrestricted recommendation in all clinical situations [3,4,5]. Furthermore, most studies are performed in vitro [5] and thus do not face the challenges of a clinical environment (e.g., patient/tongue movements and the presence of saliva/blood). A standardized methodology to measure accuracy in vivo is currently unavailable [6]; therefore, clinical trials are required to validate the efficacy of digital workflows as opposed to conventional workflows in prosthodontics.

IOS is considered to be time-saving, as the mean times for taking digital and conventional impressions of single posterior implants are reported to be 9.4–10.9 min and 14.3–15.1 min, respectively [7,8]. Furthermore, the mean measured times in the laboratory for fabricating single posterior implant crowns following digital and conventional workflows are 54.5 min and 132.5 min, respectively [9]. Ultimately, the typical side effects of taking conventional impressions, e.g., nausea or pain, as recorded by patient-reported outcome measures (PROMs), emphasize patients’ preference for IOS [1,10]. However, the acquisition costs are 10.7 times higher for digital compared to conventional impressions, which become equal after 3.6 years [11].

Clinical evaluations of the implant crowns fabricated by the respective workflows have demonstrated varying results: No adjustments at all for digital workflows, in contrast to occlusal/interproximal corrections in 30–40% for conventional workflows, were reported in [9]. Another study showed slightly more adjustments in a digital group (62.5%; 15/24 fixed dental prostheses (FDP)) versus a conventional group (61.9%; 13/21 FDP) [12]. Six and five implant crowns following digital and conventional impressions could not be inserted in a study comprising forty patients/crowns. Moreover, occlusal adjustments were necessary in 34 and 27 implant crowns, and interproximal adjustments were necessary in 28 and 15 implant crowns, respectively, in [8].

A digital workflow facilitates the outsourcing of the fabrication of restorations to a centralized (industrial) manufacturer. Furthermore, outsourcing can avoid the necessity for dental technicians to invest in expensive digital equipment [7]. However, delivery and additional time in the laboratory, e.g., the glazing or cementation of the restoration to abutments, need to be considered in a centralized workflow. Since the computer-aided design (CAD) of a monolithic implant crown takes 46.8–68 min [9], remote validation performed by only a single dental technician of an outsourced (centralized) CAD is tempting [7].

Most studies comparing both workflows have focused on time and the clinical adjustment of implant crowns at the time of insertion [7,8,13,14]. However, there is little evidence regarding outcomes after several years [15,16]. The primary aim of this randomized clinical trial was to evaluate the clinical performance of single monolithic implant crowns following a digital versus a conventional workflow. (1) The null hypothesis was that zirconia implant crowns fabricated following a digital workflow are as accurate as those obtained following a conventional workflow regarding the clinical evaluation of occlusal and proximal contacts. (2) We further hypothesized that digital and conventional impressions are equal in terms of patient satisfaction. The secondary aim was to evaluate the peri-implant health/radiographic outcomes and the Functional Implant Prosthodontic Score (FIPS) of implants restored by using the digital and the conventional workflows, respectively, at follow-up. The corresponding null hypothesis was that there is no difference in peri-implant health/radiographic outcomes (3) and the FIPS score (4) between both workflows at the last follow-up.

## 2. Materials and Methods

### 2.1. Study Design and Population

This prospective, randomized, single-blinded clinical trial involving a within-patient comparison of two workflows was conducted at the Division of Oral Surgery of the University Clinic of Dentistry, Medical University of Vienna. The study was approved by the ethics committee of the Medical University of Vienna (EK-Nr. 1108/2015) and carried out according to the principles of the World Medical Association Declaration of Helsinki (as revised in 2013).

The randomization of the study participants for allocation to the two workflows, i.e., definite insertion of the implant crown following either the digital or conventional workflows after clinical evaluation of both crowns, was performed by block randomization in blocks of 6 patients. This ensured equal-sized treatment groups for the study. Patients were allocated at the time of study participation according to a randomization list provided by a statistician. Patients were blinded to the type of implant crown, which was finally inserted after the assessment of both crowns. Concealment was not performed as the allocation of the final crown was destined a priori by the randomization list.

Thirty patients were recruited for this study according to the following inclusion criteria: (i) age ≥ 18 years, (ii) good systemic health (ASA I/II), (iii) single missing premolar/molar, (iv) healed extraction socket of ≥12 weeks, (v) adequate bone height/width at the implant site, (vi) presence of antagonistic teeth, (vii) availability for follow-up. The exclusion criteria were (i) severe systemic diseases, (ii) local radiotherapy, (iii) alcoholism/drug abuse, (iv) smoking (≥10 cigarettes/day), (v) untreated periodontitis, (vi) pregnancy, (vii) need for bone augmentation (including GBR), and (viii) antiresorptive medication. All patients needed to provide written informed consent.

### 2.2. Surgical Procedure

Before surgery, all patients were advised to rinse with 0.2% chlorhexidine for 1 min. Local anesthesia was performed by the infiltration of articaine containing 1:100,000 adrenaline (Ultracain D-S forte, Sanofi, Vienna, Austria) at the implant site. Midcrestal and intrasulcular incisions were conducted to enable raising a minimal full-thickness flap and exposing the alveolar ridge. Implant bed preparation was performed using spiral drills of increasing diameter under copious irrigation. All bone-level tapered implants (SLActive^®^, Institut Straumann AG, Basel, Switzerland) were placed according to the standard protocol of the manufacturer. Two-thirds of the implants (n = 21) had a regular platform, while one-third (n = 9) had a narrow platform. Implants revealing an insertion torque of <35 Ncm were destined for a two-stage procedure with uncovering after 3 months. Implants demonstrating a higher insertion torque were provided with a healing abutment. Sutures (Neosorb Rapid, medipac^®^, Kilkis, Greece) were removed after 7–10 days. Postoperative pain was controlled by administering 400 mg ibuprofen every 6 h. After a healing period of at least 3 months, all patients were scheduled for digital and conventional impression taking.

### 2.3. Digital Workflow (Test Group)

Digital impressions were created using an intraoral scanner (TRIOS^®^ 3, 3Shape, Copenhagen, Denmark). The healing abutment was removed, and the intraoral surfaces of the hemi-arches/complete arches were scanned. Thereafter, the scan body (CARES^®^ Mono Scanbody, Institut Straumann AG, Basel, Switzerland) was manually screwed onto the implant, and the abutment scan was recorded. After scan body removal, both the antagonist and bite registration were scanned. The scans were imported into CAD software (CARES^®^ X-Stream^TM^, CARES^®^ Visual 13, Institut Straumann AG, Basel, Switzerland). Full-contour monolithic zirconia crowns (zerion^®^ ML, Institut Straumann AG, Basel, Switzerland) with a screw-access hole were designed by the same operator and fabricated in a centralized milling facility (Etkon GmbH, Markkleeberg, Germany). The dental laboratory finalized the crowns by glazing and luting the zirconia crown onto a ti-base abutment (Variobase^®^, Institut Straumann AG, Basel, Switzerland).

### 2.4. Conventional Workflow (Control Group)

Conventional impressions were taken using an open-tray transfer coping and a polyether impression material (Impregum^TM^ Penta^TM^, 3M ESPE, Seefeld, Germany). The seating of the transfer coping was always verified by an intraoral X-ray. Impressions of the opposing jaw were taken using an alginate-based material (Alginoplast^®^, Kulzer GmbH, Hanau, Germany). Bite registration was accomplished using a vinylpolysiloxane material (Take 1^®^ Advanced Bite Registration, Kerr, Brea, CA, USA). An implant analog was fixed to the implant transfer coping. The implant model was poured in dental type V, and the alginate impression was created in a gypsum-based stone. Both were mounted in an articulator (Artex^®^, Amann Girrbach, Pforzheim, Germany) and digitized in a laboratory scanner (Ceramill^®^ Map 600, Amann Girrbach, Pforzheim, Germany). Full-contour zirconia crowns with a screw-access hole were designed by an experienced dental technician using CAD software (Ceramill^®^ Mind 3.0, Amann Girrbach, Pforzheim, Germany) and milled in house (Ceramill^®^ zirconia, Amann Girrbach, Pforzheim, Germany). The crowns were finalized by glazing and luting on the ti-base (Variobase^®^, Institut Straumann AG, Basel, Switzerland).

### 2.5. Outcomes

#### 2.5.1. Clinical Evaluation of the Implant Crown

Patients were blinded to the process of crown fabrication at delivery. One clinician with more than 20 years of experience in restorative dentistry performed the clinical evaluation. The proximal contact points were assessed using dental floss (Essentialfloss^TM^, Oral-B^®^, Procter & Gamble, Schwalbach a. Ts., Germany). A lack of mesial and/or distal proximal contacts resulted in a new crown fabrication (no crown delivery). Occlusal contacts were verified by first checking for strong contacts (Articulating paper, Dr. Jean Bausch GmbH & Co., Köln, Germany) and then checking for light contacts (Arti-Fol^®^ metallic Shimstock Film, Dr. Jean Bausch GmbH & Co., Köln, Germany). The intraorally seated crown was demonstrated to the patient in front of a hand mirror, and they were asked for their subjective opinion regarding esthetics and comfort. The decision for the final crown delivery was based on the designated crown according to randomization, clinical evaluation, and the patient’s perception.

#### 2.5.2. Patients’ Perception of the Digital/Conventional Impression Taking and the Implant-Supported Solitary Crown (Questionnaire)

All patients were invited to fill out a questionnaire after the final seating of the implant crown. The following questions addressed the satisfaction of both the workflow and the implant-supported zirconia crown: (1) preference regarding the impression technique (digital/conventional/no opinion), (2) satisfaction regarding the esthetics of the final crown (yes/no), (3) whether they would consider only digital future impressions (yes/no/maybe), (4) experience of nausea during impression taking (numeric rating scale (NRS): 1 = none to 10 = much), (5) uncomfortable clinical steps (implant placement/impression procedure/insertion of the crown/other/none), (6) the importance of posterior single tooth gap rehabilitation (NRS: 1 = none to 10 = much), (7) whether they would recommend implant-supported zirconia crowns to friends (yes/no), and (8) whether they would recommend a certain type of impression technique to friends (digital/conventional/both).

#### 2.5.3. Measurement of the Marginal Bone Level (MBL)

Radiographs recorded at the insertion of the implant crown (T_1_) and the last available recall (T_Last_) were imported into FIJI software (version: 2.9.0) [17]. The diameter of the dental implant was used for the calibration of the scale of the radiographs to maintain a consistent pixel/mm ratio [18]. Vertical linear measurements were performed from the inferior border of the beveled edge at the implant platform to the peri-implant bone on the mesial and distal aspects. Bone-level changes were denoted as the difference between T_1_ and T_Last_ of the averaged mesial/distal values. Negative values indicate a bone level inferior, i.e., bone loss, and positive values indicate a bone level coronal to the implant platform. All measurements were performed twice by the same examiner (F.B.) with >2 weeks in between (intraclass correlation coefficient (ICC): T_1_: 0.89, T_Last_: 0.82). The mean value of both measurements was used for statistical analysis.

#### 2.5.4. Evaluation of Peri-Implant Health

Implant success was defined according to Renvert et al. [19]: healthy peri-implant tissue, peri-implant mucositis, or peri-implantitis. The first was assigned if probing depths were <5 mm, there was no bleeding on probing (BoP) (except for bleeding dots), and the radiographs did not indicate bone loss >2 mm, exceeding initial bone remodeling during/after the first year. Peri-implant mucositis was defined by the presence of red, swollen tissues, profuse BoP, and/or suppuration upon probing and the absence of radiologic bone loss of >2 mm following attachment of the prosthetic supra-structure. Peri-implantitis was assigned if soft tissue inflammation corresponded to peri-mucositis and if radiographic bone loss progressed further than 2 mm after the first year following crown insertion.

#### 2.5.5. Functional Implant Prosthodontic Score (FIPS)

At the last follow-up (T_Last_), the clinical evaluation of the implant crowns was performed according to the Functional Implant Prosthodontic Score (FIPS) [20]. The score evaluated the implant crown using five variables, namely proximal contact, occlusion, design, peri-implant mucosa, and bone. Each variable is assigned a score of 0, 1, or 2, totaling a maximum FIPS score of 10 (5 × 2) per implant crown. One oral surgeon (F.B.) with more than 10 years of experience in implant dentistry rated the implant crowns by clinical evaluation, photos (lateral and occlusal), and periapical radiographs using the parallel technique.

### 2.6. Statistical Analysis

A calculation of our study’s sample size was difficult to perform due to the limited data available at the time of the initiation of this study. Using a normal distribution with a mean difference of 25 ± 20 mm, we simulated both conventional/digital impressions [21]. The power estimated by 10 k iterations of a simulation was 0.8 after accounting for a drop-out rate of 10%. The level of significance was set at α = 0.05. Randomization was performed by block randomization in blocks of 6 patients.

Normal distribution was not assumed for any tested variable. A comparison of the clinical adjustments (paired ordinal scaled variable) involved in the digital and the conventional workflows was performed using the Wilcoxon signed-rank test. The outcomes of the questionnaire and the evaluations of peri-implant health were compared using Fisher’s exact test. A test of proportion was conducted for the last questionnaire item. The agreement of MBL measurements was assessed by an ICC, and their mean was used for further analysis. Bland–Altman plots were constructed to visualize the level of agreement in terms of MBL at T_1_ and T_Last_. The difference in MBL between both groups was calculated using a permutation test. The difference in the FIPS score between both workflows was assessed using the Mann–Whitney U test. The data were analyzed according to on-treatment and intention-to-treat analysis (Supplementary Materials File S1), respectively. Data were provided either completely (analyzed) or incompletely (i.e., in case of drop-outs; not analyzed). Statistical analyses were performed using SPSS version 29 (IBM, Armonk, NY, USA) and R version 4.3.1 (R Core Team 2023).

## 3. Results

### 3.1. Patient Characteristics

**Figure 1:**
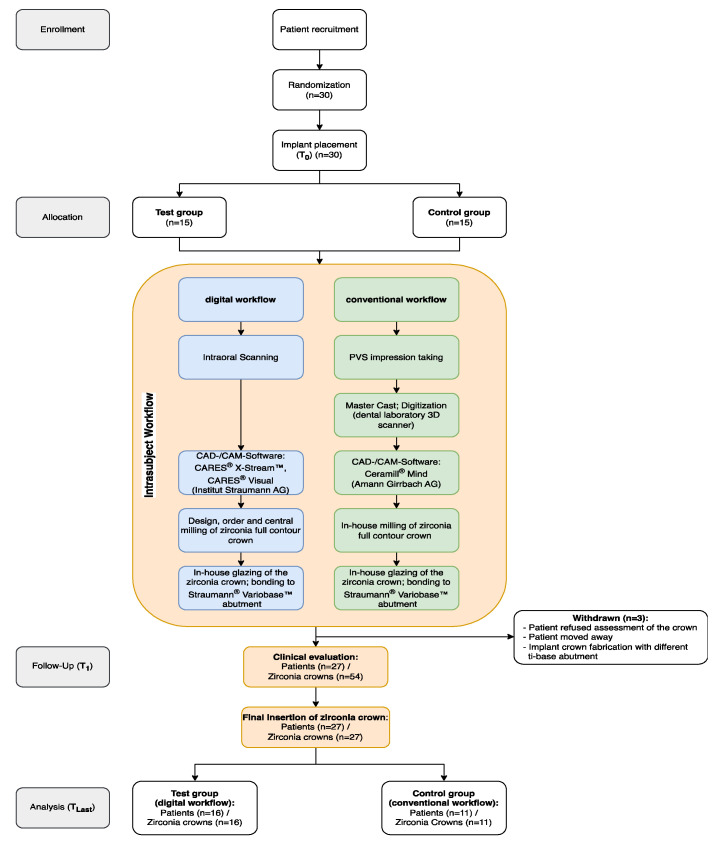
Flowchart of the study design and the clinical/laboratory procedures.

A total of 30 patients (16 females, 14 males) were initially enrolled at the time of implant placement. Three patients were considered drop-outs due to unavailability (n = 2) and implant crown fabrication with a different ti-base abutment (n = 1). A total of 27 patients (14 female and 13 male; mean age: 46.46 ± 10.55 years) with 16 implant crowns following the digital workflow and 11 following the conventional workflow were available for follow-up and included in the present analysis (Figure 1). The mean observation period between T_1_ and T_Last_ was 4.23 ± 1.10 years. The distribution of implants per site in the maxilla and mandible was 10 (PM: 9, M: 1) and 17 (PM: 4, M:13), respectively. The survival and success rates of all implants (n = 27) at the last follow-up were 100% and 96.3%, respectively.

### 3.2. Clinical Adjustment

The clinical evaluation showed that occlusal adjustments were necessary in 10 (digital workflow) and 16 (conventional workflow) out of 54 crowns. Both occlusal and proximal adjustments were performed in another 12 digital and 11 conventional implant crowns. No corrections were deemed necessary in 9.26% of 54 crowns, i.e., 5 implant crowns, following the digital workflow. There was no significant difference between the digital and conventional workflows regarding clinical adjustments (*p* = 0.334).

One crown of the digital and two crowns of the conventional workflow could not be delivered as the proximal contact point was missing and laboratory intervention was needed. Furthermore, three patients disagreed with the design of the designated crown for final insertion: two patients following the conventional workflow received a digital crown, and one patient following the digital workflow received a conventional crown.

### 3.3. Patient Perception (Questionnaire)

A total of 27 patients reported their perception of rehabilitation of a single implant site in a digital and conventional workflow. There was no distinct preference for one of the impression techniques over the other, and the patients’ preferences remain ambiguous. Furthermore, nausea during conventional impression was experienced by only 7 patients (NRS: 6–10), whereas 20 patients specified an NRS value of 1–5. There was no significant difference between the questionnaire outcomes for the groups (digital/conventional) in terms of final implant crown insertion (Table 1). Yet, more than twice as many participants recommended digital impressions to friends compared to conventional impressions (*p* = 0.095).

### 3.4. Peri-Implant Health and Technical Complications

At the last recall, 6 out of 27 implants were BoP-positive in at least one site, i.e., BoP (%) was positive in 11.11 ± 27.15 at the implant level. There was no significant difference between the implants restored following the digital or conventional workflows (*p* = 0.813). Plaque (%) at the implant level was present in 9.26 ± 26.99, demonstrating no significant difference between both workflows (*p* = 0.624). The mean PPD was 2.72 ± 0.74 mm (range: 1.0–6.0) in the digital group and 2.46 ± 0.53 mm (range: 1.0–5.0) in the conventional workflow group (*p* = 0.528). Accordingly, the peri-implant tissue of 21 implants (77.78%) was rated healthy, 5 (18.52%) demonstrated peri-mucositis, and 1 implant (3.70%) had peri-implantitis.

**Figure 2:**
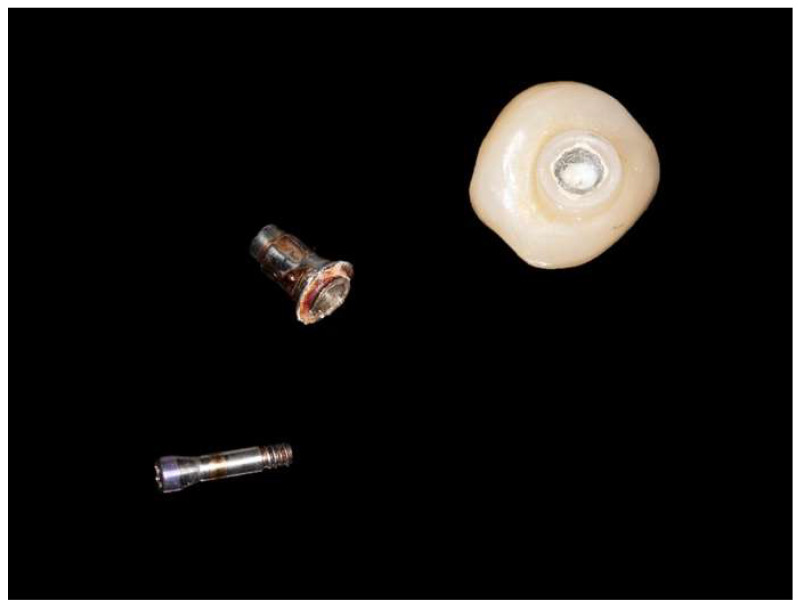
Fracture of a ti-base abutment after 3.75 years of service.

There was one fracture of a ti-base abutment (region #46) after 3.78 years following crown insertion (Figure 2). Furthermore, we observed two de-cementations of the implant crowns from the ti-base abutment.

### 3.5. Marginal Bone Level

**Figure 3:**
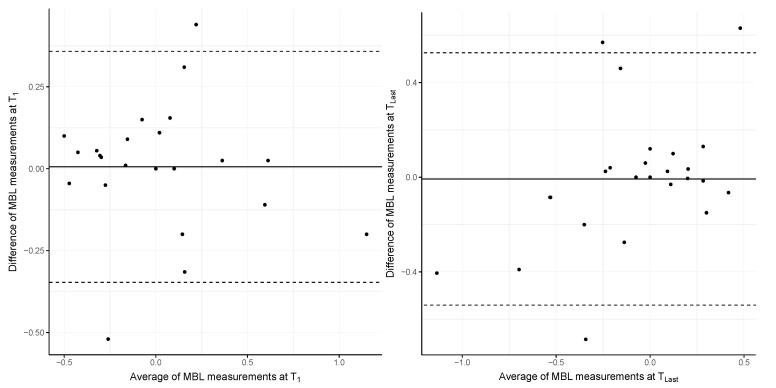
Bland–Altman plot of the MBL values of both workflows at T_1_ (limits of agreement: −0.35 to 0.36) and T_Last_ (limits of agreement: −0.54 to 0.53).

The MBL values at the time of implant crown delivery (T_1_) were −0.05 ± 0.31 mm (digital workflow) and 0.11 ± 0.43 mm (conventional workflow). At the last follow-up (T_Last_), the MBL values decreased to −0.12 ± 0.29 mm and −0.24 ± 0.57 mm, respectively. The mean overall MBL values at T_1_ and T_Last_ are depicted in Figure 3. The mean differences in MBL changes over time were 0.07 ± 0.19 mm (digital workflow) and 0.34 ± 0.62 mm (conventional workflow). There were no significant differences in bone-level changes (*p* = 0.195). Furthermore, neither group revealed any significant differences between T_1_ and T_Last_ (digital: *p* = 0.189; conventional: *p* = 0.126).

### 3.6. Functional Implant Prosthodontic Score (FIPS)

**Figure 4:**
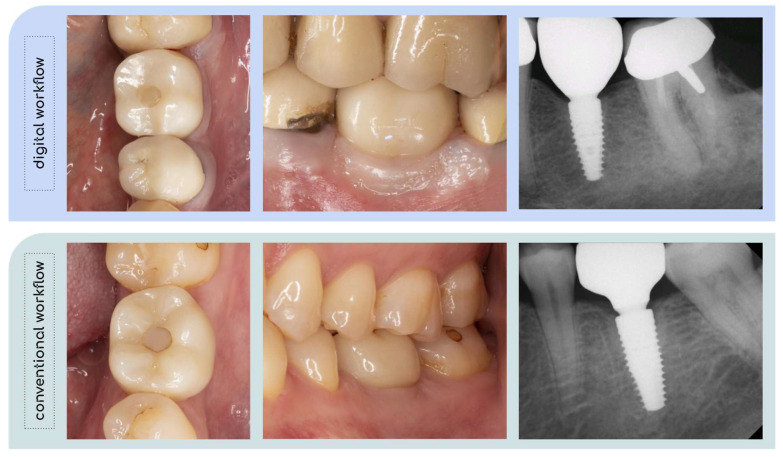
Clinical and radiographic outcomes of two study participants at the last follow-up.

The mean overall FIPS score was 8.11 ± 1.37 (range: 5–10) at the last follow-up. The mean total FIPS scores were 8.38 ± 1.15 (range: 6–10) and 7.73 ± 1.62 (range: 5–10) in the digital and conventional workflow groups, respectively. There was no significant difference between the study groups (*p* = 0.364). The highest score was observed for the variable “bone”, with a mean value of 1.93 ± 0.39, followed by “occlusion” (1.89 ± 0.32) and “design” (1.52 ± 0.7). Most minor discrepancies (i.e., a score of 1 out of 2) were found for the variables “papilla” (1.41 ± 0.64) and “mucosa” (1.37 ± 0.57). The clinical and radiographic outcomes of two study participants are depicted in Figure 4.

## 4. Discussion

This randomized controlled clinical trial aimed to evaluate clinical adjustments of implant-supported zirconia crowns and patients’ preferences with respect to following digital and conventional workflows. Furthermore, the peri-implant health/radiographic outcomes and the FIPS score were recorded and compared in both workflows at the last follow-up. Both primary and secondary aims revealed no significant differences between both workflows; thus, our hypotheses were not rejected. The implant survival and success rates indicated favorable outcomes of 100% and 96.3%, respectively. However, regarding complications, we observed two de-cementations and one fracture of the ti-base abutment.

If we relate our findings to others comparing these workflows for single posterior implant-supported crowns, it becomes evident that clinical adjustments are reported heterogeneously. One study observed no adjustments in the digital workflow compared to 14 adjustments in the conventional workflow [9]. Similar, excellent/good occlusal and proximal contact points were assessed in more than 90% and 80% of cases, respectively, requiring no corrections to monolithic zirconia (digital workflow) and ceramic veneered zirconia crowns (conventional workflow) [13]. In contrast, clinical adjustments were necessary for 15/17 monolithic LS2 cases (digital workflow) and 16/16 veneered zirconia crowns (conventional workflow) [14]. This is further supported by 36/40 (digital workflow) and 28/40 (conventional workflow) monolithic zirconia crowns necessitating chairside adjustments [8]. Our results align with the latter two studies, as all crowns except for five of the digital workflow required adjustments. Moreover, implant crowns following immediate/delayed digital and conventional impression taking required occlusal adjustments in 80/88 implant crowns (22 patients) [22]. Overall, there seems to be a tendency for fewer adjustments with the digital workflow, but the total number of adjustments and their associated time remains a concern.

The need for occlusal adjustments in crowns following the digital workflow may be linked to the alignment of the maxillary/mandibular scan(s) with the bite registration scan(s). The intersection of maxillary and mandibular scans following this alignment is a virtual phenomenon between the occluding surfaces [23,24,25]. This may impede the accurate CAD design of occlusal contacts of the implant crown. With regard to the conventional workflow, intersections were also observed in casts digitized by a laboratory scanner, although these intersections were distinctly reduced in number compared to IOS scans [25]. Furthermore, vertical deviations of the implant scan body between digital and conventional impressions, as observed in vitro, might explain the hyperocclusion of restorations derived from gypsum casts [21]. Finally, dimensional changes in the cast due to gypsum expansion may further affect the need for clinical adjustments of restorations [26].

The bone-level tapered implants demonstrated favorable results regarding healthy peri-implant tissue and the maintenance of peri-implant bone within the present observation period. At the last follow-up, the mean MBL was −0.12 ± 0.29 mm (digital workflow) and −0.24 ± 0.57 mm (conventional workflow). The mean MBL change was 0.07 ± 0.19 mm and 0.34 ± 0.62 mm, respectively. A similar peri-implant bone loss of 0.38 ± 0.24 mm was reported for bone-level tapered implants in a study with a 2-year follow-up [27]. The low change in MBL after T_1_ and the peri-implant health in 77.78% of the implants is also reflected in the overall FIPS score: 8.38 ± 1.15 (digital workflow) and 7.73 ± 1.62 (conventional workflow). A 5-year follow-up of single implant crowns following a digital workflow revealed a similar score of 8.2 ± 1.0 [28]. Furthermore, a slightly lower FIPS score was reported for the conventional workflow (6.9–7.0) compared to the digital workflow (7.2–7.9) [29], which is in line with the present study’s results. Although the MBL change was slightly higher in implants restored following the conventional workflow, the choice of treatment workflow seems to have no influence.

We also observed technical complications in the form of two de-cementations of zirconia crowns from a ti-base abutment and one fracture of a ti-base abutment within the study’s observation period. In the latter case, the patient presented with a lost implant crown, i.e., the chimney of the abutment was inside the crown; however, the remaining part of the abutment was still in situ (no fracture of the abutment screw). This seems to occur very rarely, as no fractures of ti-base abutments connected to zirconia crowns were assessed following chewing simulation and aging by thermocycling [30]. However, de-cementations have been reported despite their low incidence [15,31].

This study has limitations. First, the results, particularly those for the digital workflow (IOS: TRIOS 3, 3Shape, Copenhagen, Denmark; CAD-software: CARES^®^ X-Stream^TM^, CARES^®^ Visual 13, Institut Straumann AG, Basel, Switzerland), are valid for this specific protocol. Therefore, our comparison of the results with those of other digital protocols was limited. Second, the implant crown design was performed by different operators, namely a dental technician (conventional workflow) and one dentist (digital workflow). Hence, the effects of different operators’ CAD designs on clinical evaluations cannot be ruled out. Third, IOS scans were performed either as hemi- or as complete-arch scans, and this may have affected the virtual recording of occlusal contacts. However, a recent systematic review indicated that the accuracy of maximum intercuspation may not be impaired by hemi- or complete-arch scans [32]. Fourth, three patients received a different crown compared to the one they were randomly allocated; however, comparing the on-treatment and intention-to-treat analyses revealed only minor differences (Supplementary Materials File S1). Finally, the sample size of this study may be considered small. Thus, future studies should consider implementing either a crossover or split-mouth design for RCT to increase sample size and evaluate the effects of different workflows.

## 5. Conclusions

The clinical and radiographic outcomes of single screw-retained monolithic implant crowns are similar in both workflows, and favorable results were demonstrated after a mean follow-up of 4 years.

The digital workflow reduces the number of steps in the fabrication process for implant crowns compared to the conventional workflow, which in turn could promote higher accuracy. However, chairside adjustments were still required regardless of the workflow. Given this study’s observation period, both workflows can successfully restore posterior bone-level implants with monolithic crowns.

Regarding patients, there was no clear preference for an impression technique, although patients would recommend the digital impression more often to friends. Thus, the decision regarding the clinical workflow may be based on the patient’s and/or clinician’s preference. Future studies may address virtual intersections of digital impressions, possibly reducing the need for clinical adjustments. Furthermore, the CAD design of crowns might benefit from artificial intelligence, as this process remains time-consuming regardless of the choice of workflow.

## Figures and Tables

**Table 1:** Outcomes of patients’ perception of single missing tooth rehabilitation following a digital/conventional workflow.

Question	Outcome																								*p*-Value
(1) Preference of impression technique	Digital (n = 9)								Conventional (n = 7)								No opinion (n = 11)								0.375
(2) Satisfaction with implant crown’s esthetics	Yes (n = 26)												No (n = 1)												0.407
(3) Perform future impressions digitally	Yes (n = 10)						No (n = 2)						Maybe (n = 2)						n/a (n = 13)						0.162
(4) Nausea during conventional impressions (NRS)	1 (n = 13)				2 (n = 2)				3 (n = 4)			5 (n = 1)			6 (n = 4)			7 (n = 2)			8 (n = 1)				0.372
(5) Discomfort in clinical step(s) (from implant placement to insertion of the crown)	None (n = 22)				Analog impression (n = 1)				Insertion of crown (n = 1)				Implant placement/ Impression (n = 1)				Implant placement/ impression/ insertion of crown (n = 1)				Other (n = 1)				0.316
(6) Importance of single posterior tooth gap rehabilitation (NRS)	1 (n = 2)					5 (n = 4)					8 (n = 1)					9 (n = 2)				10 (n = 18)					0.888
(7) Recommendation of implant-supported zirconia crowns to friends	Yes (n = 26)												No (n = 1)												0.407
(8) Type of impression technique recommended to friends	Digital (n = 16)						Conventional (n = 7)						Both (n = 3)						None (n = 1)						0.503

n represents the absolute number of valid answers contributing to our statistical analysis; NRS = numeric rating scale.

## Data Availability

The data are not publicly available due to privacy restriction.

## Supplementary Materials

The following supporting information can be downloaded at: https://www.mdpi.com/article/10.3390/jcm13020432/s1, File S1: Results following an intention-to-treat analysis.
