# Supplementary material for: Clinical and Radiographic Outcomes of Single Implant-Supported Zirconia Crowns Following a Digital and Conventional Workflow: Four-Year Follow-Up of a Randomized Controlled Clinical Trial
Source: J Clin Med. 2024 Jan 12;13(2):432. doi: 10.3390/jcm13020432 (PMC10816133; doi:10.3390/jcm13020432)
Supplement: Supplementary file 1 [file jcm-13-00432-s001.zip › jcm-2782753-supplementary.pdf]

File S1. Results following an intention-to-treat analysis

Distribution according to initial treatment allocation:
Analyzed: digital workflow (n=15), conventional workflow (n=12)

3.3 Patient's perception (questionnaire)

Question		p-value
**(1) Preference of impression technique**		0.715
**(2) Satisfaction with implant crown's esthetics**		0.444
**(3) Perform future impressions digital**		0.454
**(4) Nausea during conventional impressions (NRS)**		0.643
**(5) Discomfort of clinical step(s) (from implant placement to insertion of the crown)**		0.388
**(6) Importance of single posterior tooth gap rehabilitation (NRS)**		0.811
**(7) Recommendation of implant-supported zirconia crowns to friends**		0.444
**(8) Type of impression technique recommended to friends**		0.108

3.4 Peri-implant health and technical complications

Parameter	Digital Workflow	Conventional Workflow	p-value
**BoP (%)**	-	-	1.000
**Plaque (%)**	-	-	1.000
**PPD**	2.68 ± 0.77 mm (range: 1.0-6.0)	2.52 ± 0.53 mm (range: 1.0-5.0)	0.990

3.5 Marginal bone level

Parameter	Digital Workflow	Conventional Workflow	p-value
**MBL (T_1)**	-0.11 ± 0.26 mm	0.16 ± 0.43 mm	-
**MBL (T_{Last})**	-0.14 ± 0.3 mm	-0.21 ± 0.55 mm	-
**MBL (T_1-T_{Last})**	0.03 ± 0.14 mm	0.37 ± 0.59 mm	p=0.105
**MBL (T_1-T_{Last})$_{Digital\ Workflow}$**	-	-	p=0.365
**MBL (T_1-T_{Last})$_{Conv.\ Worfklow}$**	-	-	p=0.09

3.6 Functional Implant Prosthodontic Score (FIPS)

Paramter	Digital Workflow	Conventional Workflow	p-value
**FIPS Score (mean)**	8.4 ± 1.18 (range: 6-10)	7.75 ± 1.55 (range: 5-10)	p=0.315
